# Mass Spectrometric Screening of Ovarian Cancer with Serum Glycans

**DOI:** 10.1155/2014/634289

**Published:** 2014-02-04

**Authors:** Jae-Han Kim, Chang Won Park, Dalho Um, Ki Hwang Baek, Yohahn Jo, Hyunjoo An, Yangsun Kim, Tae Jin Kim

**Affiliations:** ^1^ASTA Inc., Gyeonggi Biocenter, Eui-dong, Youngtong-gu, Suwon 443-270, Republic of Korea; ^2^Department of Food and Nutrition, Chungnam National University, Yuseong-gu, Daejeon 305-764, Republic of Korea; ^3^NosQuest Inc., U-Space 1A-dong 1103, Bundang-gu, Seongnam 463-400, Republic of Korea; ^4^Hudson Surface Technology Inc., 180 Old Tappan Road, Building 1, Suite 3, Old Tappan, NJ 07675, USA; ^5^Graduate School of Analytical Science and Technology, Chungnam National University, Yuseong-gu, Daejeon 305-764, Republic of Korea; ^6^Department of Obstetrics and Gynecology, Cheil General Hospital and Women's Healthcare Center, Kwandong University, College of Medicine, Mukjeong-dong, Jung-gu, Seoul 100-380, Republic of Korea

## Abstract

Changes of glycosylation pattern in serum proteins have been linked to various diseases including cancer, suggesting possible development of novel biomarkers based on the glycomic analysis. In this study, N-linked glycans from human serum were quantitatively profiled by matrix-assisted laser desorption ionization time-of-flight (MALDI-TOF) mass spectrometry (MS) and compared between healthy controls and ovarian cancer patients. A training set consisting of 40 healthy controls and 40 ovarian cancer cases demonstrated an inverse correlation between *P* value of ANOVA and area under the curve (AUC) of each candidate biomarker peak from MALDI-TOF MS, providing standards for the classification. A multibiomarker panel composed of 15 MALDI-TOF MS peaks resulted in AUC of 0.89, 80~90% sensitivity, and 70~83% specificity in the training set. The performance of the biomarker panel was validated in a separate blind test set composed of 23 healthy controls and 37 ovarian cancer patients, leading to 81~84% sensitivity and 83% specificity with cut-off values determined by the training set. Sensitivity of CA-125, the most widely used ovarian cancer marker, was 74% in the training set and 78% in the test set, respectively. These results indicate that MALDI-TOF MS-mediated serum N-glycan analysis could provide critical information for the screening of ovarian cancer.

## 1. Introduction

Protein glycosylation is one of the most important posttranslational modifications, resulting in the attachment of glycans (carbohydrate chains) to proteins. Glycans have been reported to be associated with critical functions of proteins and to be involved in many biological processes such as cell signaling, extracellular interactions, infections by pathogens, immune responses, and pathogenesis of cancer [1]. One of the major types of glycans is the N-linked glycans, which are connected to asparagine amino acid residues of proteins, via the amide nitrogen of asparagine [2]. Most of the membrane proteins and secreted proteins are glycosylated by hundreds of glycosyltransferases in endoplasmic reticulum and Golgi apparatus [3]. N-linked glycans, which could be released from glycoproteins by an enzyme, peptide-N-glycosidase F (PNGase F) [4], have been detected in human serum proteins such as immunoglobulins [5].

Alteration of glycosylation patterns in cell lines, sera, or tissue samples from cancer patients has been identified, and numerous kinds of cancer, including prostate, ovarian, breast, and gastric cancer, demonstrated modification in the overall or specific glycosylation patterns [6], indicating that glycans could be employed as useful cancer biomarkers. In addition, most of currently approved cancer biomarkers such as CA-125 or CEA are well known glycoproteins [7, 8], of which quantitative and qualitative changes directly lead to altered patterns of oligosaccharide chains. It is now believed that increased activities of extracellular matrix metalloproteinases or immune responses in cancer patients also contribute to altered glycan patterns in circulation [9]. Therefore, human serum glycome has indeed great potential to provide enormous amount of valuable information regarding carcinogenesis and other pathogenesis.

Recent technical progress provided many valuable tools, in particular mass spectrometry, to study glycomes, the entire profiles of glycans in biological systems, leading to possibilities of disease biomarker development through glycomic analysis [6]. Use of solid phase extraction (SPE) with graphitized carbon cartridges (GCC) successfully isolates oligosaccharides from proteins, salts, and other contaminants after the release of glycans from proteins by PNGase F [10]. Additionally, elution with different concentrations of acetonitrile/H_2_O significantly increased the isolation efficiency of minor species of glycans in the sample, leading to better quantitative detection of glycans which existed in smaller fraction of total glycan population [11]. These enhanced procedures for the analysis of glycomes eventually enabled mass spectrometry profiling of total glycomes in natural forms without chemical modifications like derivatization, and ion suppression by major glycans was limited to a level where a broad spectrum of glycans could be detected and quantitatively analyzed [12]. These mass spectra of glycans could be employed as multibiomarker panels from which quantitative behavior of each glycan molecules can be traced and compared for the purpose of disease screening. When combined in an optimized manner, these multiple biomarkers usually led to far better diagnostic classifications than single-marker approaches [13]. Furthermore, glycan profiles are far less complicated than proteomic analysis since theoretically ~1600 N-linked glycans were predicted and less than 200 N-glycans are actually detected in the human serum [14], suggesting less number of parameters for the classification analysis and still sufficient amount of information for accurate differentiation of sample sources. Well-curated glycan libraries or databases such as GlycoMod [15] and GlycoSuiteDB [16] are easily available and help correct annotations of glycan ion signals in MALDI-TOF MS spectra, leading to improved characterization of glycomes [14]. With these advantages integrated, a couple of studies regarding correct prediction of cancer patients based on mass spectrometry analysis of human serum glycomes were reported [10, 17], highlighting the promise of better cancer screening with MS-mediated glycomic analysis.

CA-125 is currently the most common tumormarker for ovarian cancer [18], but its use for the detection of ovarian cancer at an early stage is very limited since CA-125 level is elevated only in ~50% of stage I ovarian cancer [19]. Considering that ~19% of ovarian cancer cases are first detected at stage I with 5-year survival rate ~93% and that majority of ovarian cancer patients (67–74%) are diagnosed at much later stages (stages III and IV) mostly with metastatic status [20], it is of critical importance to develop biomarkers which can provide reliable information for the early screening of ovarian cancer. CA-125 is mostly used for the monitoring of ovarian cancer after medical treatment.

In this study, we attempted to develop a multibiomarker panel which could classify ovarian cancer patients from healthy controls on the basis of MALDI-TOF MS analysis of human serum N-glycans. N-linked glycans were isolated from patients' sera as well as from healthy controls' sera and analyzed by MALDI-TOF MS, generating signals from multiple biomarkers containing glycans. These MALDI-TOF MS biomarkers were quantitatively analyzed to discriminate ovarian cancer patients from healthy controls in a training set. Biomarkers were selected on the basis of the correlation between *P* values of ANOVA and AUC of individual biomarkers, leading to a screening system for the ovarian cancer. We further evaluated this screening system on a blind test set in comparison with the classifications with serum CA-125 levels.

## 2. Materials and Methods

### 2.1. Informed Consent and Approval by an Ethics Committee for the Collection of Human Samples

Individual serum samples were acquired from patients with informed consent in Kwandong University College of Medicine, Cheil General Hospital & Women's Healthcare Center, Seoul, Republic of Korea, according to the protocol approved prior to the initiation of this study by the Institutional Review Board (IRB) of Cheil General Hospital & Women's Healthcare Center.

### 2.2. Serum Sample Collection

The diagnosis of ovarian cancer and determination of the FIGO (International Federation of Gynecology and Obstetrics) stages [21] were carried out at Cheil General Hospital & Women's Healthcare Center. Whole blood was collected into a tube, followed by clot formation at room temperature. Then, the samples were centrifuged at 1000 ×g for 10 minutes at room temperature. The supernatants were carefully transferred into clean polypropylene tubes (Eppendorf microfuge tubes, 1.5 mL) and serum CA-125 level was measured by using the electrochemiluminescence immunoassay technique and an Analytics E170 (Elecsys module) immunoassay analyzer (Roche Diagnostics GmbH, Mannheim, Germany). Eventually, serum samples were stored in aliquots of ~100 *μ*L at –80°C.

### 2.3. Isolation of N-Linked Glycans

Fifty microliter of a serum was mixed with 50 *μ*L of 200 mM NH_4_HCO_3_ (Sigma-Aldrich, A6141) containing 10 mM of dithiothreitol. A moderate protein denaturation was carried out with 4 cycles of alternating 15 seconds in boiling water and 15 seconds in a water bath at room temperature, for 2 min in total. N-linked glycans were released from denatured proteins by the addition of 1000 units of peptide-N-glycosidase F (PNGase F, New England Biolabs, P0705). The PNGase F reaction was carried out in a microwave-mediated enzyme reaction enhancing system (REDS, HST, SPM0201000) for 10 min at 37°C with 400 W of microwave power output. Then, ice-cold ethanol (400 *μ*L) was added, and proteins were precipitated in −80°C freezer for 1 hr. Protein precipitates were removed after centrifugation and the supernatant carrying N-linked glycans was transferred to new tubes and dried in an evaporation system (EZ-2, Genevac). Solid phase extraction (SPE) of N-glycans was performed with graphitized carbon cartridges as described previously [12]. Before loading, cartridges were washed and equilibrated with 0.1% trifluoroacetic acid (TFA) in 80% acetonitrile/H_2_O. Glycan solutions were applied to the cartridges that were washed subsequently with Nanopure water. Glycans were eventually eluted in 10% acetonitrile (ACN)/H_2_O, 20% ACN/H_2_O, and 40% ACN/H_2_O with 0.05% TFA, in a sequential manner. Each fraction was collected and fast-dried in an evaporator. Dried glycans were dissolved in 15 *μ*L of purified water. The acidic glycan fraction (40% ACN/H_2_O) was not used to discover the biomarkers since biomarkers from 10% and 20% fractions gave analytical information sufficient enough for the development and evaluation of multibiomarker panel for the diagnosis of ovarian cancer temporarily. The 40% fraction will be analyzed with possible modifications such as permethylation in the following studies.

### 2.4. Mass Spectrometric Analysis

The glycan solution (1 *μ*L) was placed onto a sample spot of the 2, 5-dihydroxybenzoic acid (DHB)-prespotted MALDI target plates (HST, PFA2317DN0, 1700 *μ*m spot size) and mixed with 1 *μ*L of 70% ACN/12 mM NaCl solution. Loaded sample mixture was dried fast in vacuum. All mass spectra were acquired by 4800 Plus MALDI TOF/TOF analyzer (AB SCIEX). Glycan fractions of 10% and 20% ACN were analyzed in the positive-ion reflectron mode, and an *m/z* range from 1000 to 3000 was monitored. Twenty-five laser shots were pulsed on a position, and data were collected from 40 positions on an individual sample spot (total laser shots were 1000 on a sample spot). Mass calibration was based on the twelve internal calibrants involved in N-glycans with 0.2 *m/z* tolerance. Four multiple spectra were obtained from each fraction (10% or 20% ACN/H_2_O) of a single sample.

### 2.5. Analysis of MS Data

Data Explorer 4.5 (Applied Biosystems) and an in-house software package were used for the extraction of ion peak information from MS spectra. The in-house software package simply transferred the entire information of peaks (centroid mass values, the signal-to-noise ratios, heights, etc.) from a spectrum onto a tabulated data format such as MS Excel. Centroid *m/z* values with absolute ion peak intensities were tabulated and subsequently arranged in an order for following statistical analysis. The signal-to-noise ratio for the extraction of peaks was 4, and threshold for the selection of peaks was newly set to 10.

### 2.6. Normalized Absolute Peak Intensity

Normalized absolute peak intensity (NAPI) was calculated as follows:

(1) $$\begin{array}{c} \text{NAP}{\text{I}}_{i}=\frac{\text{AP}{\text{I}}_{i}}{\sum_{j=1}^{n}\text{AP}{\text{I}}_{j}}, \end{array}$$

where *n* is the number of peaks in a mass spectrum. Each absolute peak intensity (API) was divided by the sum of total API in the spectrum including isotopes. For the convenience of calculation, each NAPI was multiplied by 1,000.

### 2.7. Statistical Analysis

MS Excel 2007 (Microsoft), Sigmaplot 10.0 (Systat), SigmaStat (Systat), and MATLAB 7.0 (Mathworks) were used for the marker discovery and statistical analysis. The possible glycan composition was annotated by the GlycoMod (http://ca.expasy.org/tools/glycomod/). The search was performed within the mass tolerance of ±10 ppm for 10% and 20% fraction data.

### 2.8. Determination of Biomarkers

Biomarkers were selected by the comparison of patients and healthy controls using the intensities of monoisotopic peaks. Initially, monoisotopic peaks with the *P* value smaller than 10^−12^ from ANOVA were selected as potential biomarkers. Then, the receiver operating characteristic (ROC) curve of each potential biomarker was calculated in the training set to decide the classification efficiency of biomarkers. Eventually, the peaks with the area under the curve (AUC) of ROC curve greater than 0.72 (10% ACN/H_2_O fraction) and 0.75 (20% ACN/H_2_O fraction) were chosen as the multibiomarker set for further evaluation in the blind test set.

### 2.9. Screening and Classifications

Multiple biomarkers have been used for the screening of samples. Since we employed the multiple biomarkers for the diagnosis, the classification efficiency of each biomarker needs to be integrated into the final classification algorithm. Classification cut-off values of individual biomarkers were determined on the basis of their sensitivity values in the training set. From the ROC curve of each biomarker, a cut-off value was deduced at the specific sensitivity of biomarkers. Using 15 biomarkers selected from the training set, each sample, in return, received prescreening information about the intensities of those 15 *m/z* peaks from the mass spectra. Using the cut-off values obtained from ROC curves of biomarkers, the ion peak intensity information of individual biomarker, either from patients or from healthy controls, was converted to a score of +1 and −1 according to the classification as a positive for ovarian cancer or as a negative as far as the individual biomarker. Since the efficacy of each biomarker was different, the score of ±1 for the screening was then multiplied by weighting factors which were the AUC of each biomarkers. Then, the total score from the 15 biomarkers of a serum sample was used to classify the sample. The diagnostic cut-off values of the total score were determined in the training set as well.

## 3. Results

### 3.1. Sample Information

Collected human serum samples were divided into two sets: one was the biomarker discovery set for the establishment of classification standards (training set), and the other blind test set was for the independent validation of the biomarkers and classification standards. A training set composed of 40 healthy controls and 40 ovarian cancer patients was selected from a total of 63 healthy controls and 77 ovarian cancer patients in a random manner except for the age (Table 1). In order to minimize the biological bias other than disease status, identical number of samples from each bracket of age was selected (Table 1). Average ages of patient and control groups were 41.8 ± 9.5 and 41.2 ± 9.7, respectively. Age was regarded as a parameter which could contribute to the analysis as a covariate, and we attempted to minimize the influence of age factors on the biomarker discovery by matching the ages as much as possible between the patient group and healthy controls. Patient serum samples varied in the cancer stages (Table 2) and cell types. Among 40 ovarian cancer patients for the training set, 19 samples were at stage I of ovarian cancer, and 3 samples were at stage II, both of which were considered as early-stage samples in this study. The number of patients at stage III and stage IV was 15 and 3, respectively (Table 2). After the selection of the training set, the rest of the controls and patients comprised a blind test set. All the patients and healthy controls were FarEast Asians, specifically Koreans. There was no study subject whose ethnic origin was not Korean in this study.

### 3.2. MALDI-TOF Mass Spectrometry

N-glycans purified from serum samples were subjected to MALDI-TOF mass spectrometry. For each serum sample, we used 10% ACN and 20% ACN fractions of N-glycans for this study. Each fraction of N-glycan sample was analyzed four times (four independent target spots) by MALDI-TOF mass spectrometry, generating a quadruplicate of mass spectra from a fraction per sample, and all 8 of the mass spectra (10%, 20% fractions) from a serum sample have been used for the data analysis and classifications. Although the serum processing was focused on the N-glycans, we detected many ion peaks which were not annotated by glycan libraries or databases. These supposedly nonglycan ion peaks implicated that there were a lot of mass peaks for molecules other than N-glycans in the mass spectra. We did not excluded those nonglycan mass peaks from the biomarker discovery analysis since quantitative difference of a biomolecule in the serum could contribute significantly to disease screening whether the ion peaks were derived from N-glycans or not.

The % CV (coefficient of variation) of the peak intensities which describes the quantitative reproducibility was smaller than 15% throughout the analysis. Each sample was analyzed in a set of 4 different MALDI-TOF MS spots (a quadruplicate), and the average % CV of all peaks obtained from these quadruplicates in the entire set of mass spectra was maintained under 15%, which seems to show the quantitative integrity of this analysis. In a similar analysis of sample preparation and MALDI-TOF MS, % CV of all ion intensities was 7.28% with absolute intensities across quadruplicate spots [22].

### 3.3. Marker Discovery in the Training Set

**Figure 1:**
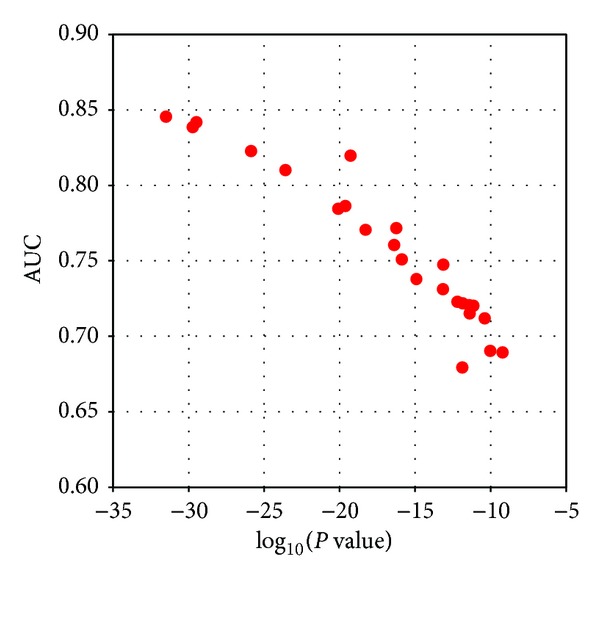
Relationship between AUC and *P* value of putative biomarkers. The AUC of a putative biomarker and its *P* value of ANOVA obtained from the training set were plotted to show the relationship between them.

The mass spectrometric data from the training set were integrated with regard to *m/z* values and corresponding intensities in the mass spectra. Peak intensities were normalized with total ion abundance of a mass spectrum. The difference of mean peak intensities between healthy controls and ovarian cancer patients was analyzed by ANOVA, providing *P* values for each *m/z* peak. The mass peaks showing *P* values smaller than 10^−9^ and the frequency higher than 95% in both patient and control groups were selected as potential biomarkers. A total of 24 potential biomarkers had *P* values under 10^−9^ and detection frequency over 95% (Table 3). The *m/z* values were the average observed mass across the sample set. The standard deviation of observed mass values and the mass errors against the theoretical mass retrieved from public databases were derived from the mass spectra of the training set (Table 3). About half (10/24) of them were annotated as N-glycans with defined compositions while 6 other glycan molecules were included in the list of potential biomarkers (Table 3). After prescreening of potential biomarkers, we calculated the area under the curve (AUC) for each potential biomarker from the receiver operating characteristic (ROC) curve which was generated by peak intensity data from the training set. Specificity and sensitivity for the ROC were derived from the training set. As shown in Figure 1, AUC and the log *P* value exhibited inverse linear correlations, indicating that both AUC and *P* values were important parameters for the selection of effective biomarkers. We arbitrarily chose AUC 0.72 as a threshold for a biomarker from 10% ACN fraction (5 peaks), and biomarkers for 20% ACN fraction were selected with *P* values below 10^−15^ as well as with AUC over 0.75 (10 peaks) (Table 3). Thus, 15 candidate biomarkers from MALDI-TOF mass spectrometry comprised the multibiomarker panel for this study. All of 5 markers from 10% fraction were assigned as glycans, and 5 out of 10 peaks in 20% ACN fraction were also assigned as glycans. Other markers were not assigned as glycans composed of hexose, N-acetylhexosamine, fucose, and sialic acids. However, we included those nonassigned peaks in further analysis because they exhibited important aspects of biomarkers such as significant differences in the peak intensities with high AUC and almost universal detection in all the mass spectra generated from patient and control samples.

### 3.4. Classification with the Multiple Biomarker Panel

The classification efficiency of the biomarkers selected on the basis of the training set was evaluated by AUC of receiver operating characteristics (ROC) curves. Each biomarker had its own cut-off values which were decided by standard specificity or sensitivity from its own ROC curves based on the training set. With a combined multibiomarker panel, we devised a scoring system to put together the information from multiple biomarker peaks. The screening score is calculated with the basic classification values (+1 or −1) determined by the cut-off threshold. The score is further sophisticated by a weighting factor proportional to the classification efficiency of the biomarker peak intensity between ovarian cancer patients and healthy controls. Standard sensitivities such as 70% or 80% were applied to the training set, eventually leading to 0.83 or 0.85 of AUC, respectively, with 5 biomarker peaks from 10% fraction. Standard sensitivities were used for the determination of the cut-off value in the ROC curve of a MS peak biomarker, and then the cut-off values of each biomarker peak were used to determine a score from a peak in a mass spectrum. The sum of the score from biomarker peaks in a spectrum was collected in the training set and used as an eventual variable to make ROC from the initial standard sensitivity. The combination of 10 candidate biomarker peaks from 20% fraction resulted in 0.88 of AUC when the threshold was set by standard sensitivity of 70% in the training set (data not shown).

**Figure 2:**
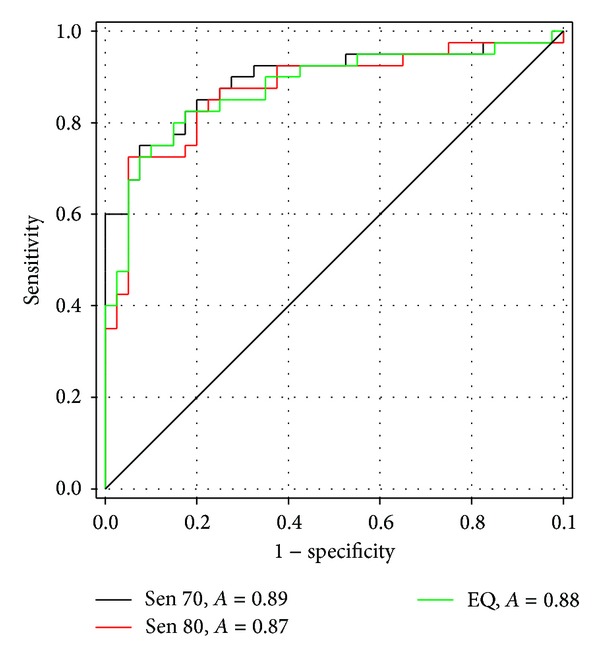
ROC curves of the classification in the training set. ROC curves were derived with the combination of biomarkers from both 10% ACN and 20% ACN fractions. The cut-off threshold of each biomarker was set to meet the standard sensitivities (below the plot). A scoring system with weighting factors based on AUC of each biomarker integrated information from multiple biomarkers, providing ROC curves. Standards for the determination of the cut-off values were displayed below the plot (Sen70, sensitivity 70%; *A*, AUC, Sen 80, sensitivity 80%; EQ, sensitivity = specificity).

When all the 15 biomarker peaks both from 10% and from 20% fractions were integrated, the AUC was improved to 0.89 when the standard sensitivity 70% for the cut-off values was applied to the training set (Figure 2). The scoring system with 15 biomarker peaks provided different sensitivities and specificities for the training set when the thresholds for the screening score were changed (Table 4). The training set resulted in 80~90% sensitivity with specificity ranging 70~83%, whereas sensitivity obtained by CA-125 (35 U/mL as a cut-off value) was 74%, mostly short of that from the panel of MS peak biomarkers.

### 3.5. Evaluation of Predicting Power of the Panel of Biomarkers

To evaluate the multiple biomarkers developed from the training sets, a blind test of 60 unknown samples was performed. Unknown test set was composed of 23 healthy controls and 37 ovarian cancer patients (Table 5). The age of healthy controls was between 20 and 60, but the 37 ovarian cancer patients were diverse in age from 30 to 89. With different screening threshold scores, sensitivity and specificity for the blind test set were calculated by using 15 biomarkers and the scoring system developed with the training set. The cut-off thresholds for individual biomarker peak was derived with the standard sensitivity of 70% in the training set, and the screening cut-off scores used in the training set were applied to the blind test set to evaluate the predicting capability. The specificity remained constant at 83% with different score thresholds, while sensitivity varied from 81% to 84%, depending on the score thresholds (Table 6). The sensitivity by the serum level of CA-125 in the blind test set was 78%, which was still short of those obtained by the MS peak biomarker panel of this study from MALDI-TOF mass spectrometry of serum glycans.

The screening system developed from the training set was applied to another set of ovarian cancer patients who experienced recurrence after initial surgery for the ovarian cancer (see Supplementary Table 1 in Supplementary Material available online at http://dx.doi.org/10.1155/2014/634289). In this recurrent set, all the 8 patients were successfully classified as patients regardless of cell types and stages. Among patients with measurements of the serum CA-125 level in the recurrent cases, only 60% (3 cases) were above the conventional cut-off value 35 U/mL. However, it should be noted that the sample number of recurrent cases was very small (*n* = 8) to make any valid conclusions about the superiority of biomarkers at this point.

When the histological types of ovarian cancer were taken into account, clear-cell type ovarian cancer revealed impressive sensitivities from serum glycan MS analysis, better than those from CA-125 by as much as 20% (Supplementary Table 2) in both the training set and blind test set. The serous type of ovarian cancer also demonstrated sensitivities of glycan analysis slightly better or just similar to those from CA-125, implicating that there could be varieties in the classification efficiency due to the types of ovarian cancer as is the case with CA-125. There should be further analysis regarding the relationship between cell types and classification efficiencies since the current set of subjects is relatively small and may be insufficient to determine general tendencies. Mucinous types of ovarian cancer had only 2 cases included in the blind test set and were not included in the analysis of the effect of cell types.

## 4. Discussion

In the MALDI-TOF MS analysis of human serum N-glycans, we developed a multibiomarker panel for the screening of ovarian cancer patients and established a screening system based on the biomarker peak intensities showing quantitative differences between cancer patients and healthy controls. The screening system demonstrated classification capability both in the training set itself as well as in the blind test set where the predictive power of the biomarker peaks was evaluated for potential clinical application. When compared with CA-125, the most common ovarian cancer biomarker in the clinical practice, the screening system using MALDI-TOF MS biomarkers resulted in improved performance of classifications both in the training set and in the blind test set.

The correlation between the *P* values from ANOVA and the AUC (area under the ROC curve) which show the authentic effectiveness of biomarkers was investigated in this study. *P* values of ANOVA are frequently referred to for the selection of potential biomarkers. It has been expected that *P* values and AUC are closely associated, but we systematically made a plot showing their relationship in an analytical manner. Moreover, in this study, the diagnostic efficacy was validated in an independent set of 60 blind samples (23 healthy controls and 37 patients) that had been randomly selected. The classification rate was evaluated in this blind test set with biomarkers selected from the training set. To avoid the problem of “overfitting,” this type of cross-validation in the blind test set is essential. In addition, we would like to put an emphasis on the fact that our classification system developed in this study was quite efficient in the identification of recurrent ovarian cancer patients.

In this study, we rather focused on the diagnosis than the marker discovery. Though we followed the protocols that have been previously published, we intended to use every *m/z *signal which satisfied the following conditions that (i) isotopic peak distribution was apparent; (ii) peaks were observed throughout the samples in a given analytical condition; and (iii) peaks were strong and quantitatively stable enough to distinguish the patients and healthy control groups. Although some of the markers did not match with theoretical mass values of glycans in databases, we insisted to use those peaks because they were consistently detected in almost every sample including the blind test set, and their intensities were stable and reproducible enough to minimize the concerns regarding the quantitative interference by the nonglycan peaks on the overall peak intensities. Regardless of molecular characteristics of biomarkers, we pursued the clinical values of disease markers in order to develop a practical diagnostic system out of mass spectrometric analysis of serum biomolecules.

N-glycans attached on the serum proteins are a diverse source of physiological and pathological information. Their structure and quantity have been reported to reflect the biological condition of the serum's host. The consequence the nature of N-glycan alterations is not completely understood yet. Nevertheless, it is clear that the N-glycan profiles at a certain moment can be an indication of pathogenesis. Biomarkers can be discovered by the comparison of a patient group to a nonpatient group trying to find the signal that can possibly distinguish the two groups. Thus, even though we do not understand the biological context of changes, nor the exact identity of the mass spectrum ions, the mass spectrometric signals that exhibited apparent differences between two groups should be exploited for better screening methods. Five out of 15 MS peak biomarkers in this study were unable to be assigned as N-glycans according to their accurate masses. Interestingly, those unassigned markers were consistently observed throughout the patient and healthy control samples with different intensities. Furthermore, they were shown in the repetitive analysis of training sets as well as in the blind test set, which suggested that those unidentified molecules should be authentic components of human serum. In this prospective, though the nature of unassigned markers was not elucidated, they were included as a part of biomarkers because they were able to distinguish the patient samples.

While the peak intensities from two groups were compared, *P* values obtained from ANOVA has been used to determine the potential biomarkers due to the simplicity of the method. However, *P* value itself cannot be a parameter that represents the efficacy of each marker. To search for the effective biomarkers with simple ANOVA, we investigated the correlation between *P* values and AUC from ROC curve. As shown in Figure 1, log_10_ (*P* value) was linearly correlated to the AUC that represents the discriminating potential of the biomarkers. Considering the fact that ANOVA is one of the convenient methods to compare the difference between two groups, the correlation of *P* values to AUC suggests a tentative threshold for further discovery of biomarker.

The validity of biomarkers was evaluated by the classification rate in the blind test set. Among the 60 blind serum samples that included 23 healthy women and 37 ovarian cancer patients at various stages, we could discriminate patients with 84% of sensitivity and 83% of specificity. There is little variation in the specificity of the blind set along the different score thresholds, suggesting relatively stable range of classification thresholds. Though there were small changes in the sensitivities of serum glycan analysis, it should be noted that the sensitivities derived from serum glycan analysis were always higher than those from CA-125 while maintaining specificity at 83% in the independent validation set. This emphasizes that MALDI-TOF MS analysis of human serum glycans could be developed into highly efficient screening system for ovarian cancer patients. Another possibility of developing an efficient screening system for ovarian cancer would be the combination of the CA-125 measurement with MALDI-TOF MS analysis of serum glycans. We have tested this possibility by combining the MALDI-TOF MS analysis of serum glycans with CA-125 test, and such combination with CA-125 cut-off 35 U/mL significantly enhanced the sensitivity of the results (~15%, data not shown) when a sample was categorized as a patient's sample either by the serum glycan profiling or by CA-125 test. However, due to the small size of the sample set (~41) and the lack of complete information of CA-125 for each subject in the set, it would be premature to make valid conclusions concerning the potential of combining the serum glycan profiling by MALDI-TOF MS with CA-125 test.

Serum glycan analysis based on MALDI-TOF MS also resulted in the complete detection of ovarian cancer patients with recurrence (Supplementary Table 1). If confirmed with more recurrent ovarian cancer patients in a larger cohort, serum glycan analysis may also be used for the monitoring of the ovarian cancer after primary medical treatment or for the prognosis of the disease.

By analyzing the N-glycome of human serum with MALDI-TOF mass spectrometry, we developed a multibiomarker panel consisting of MALDI-TOF MS ion peaks that enabled the screening of ovarian cancer with high accuracy, and a screening system utilizing the intensity differences and weighting factors was also established. The actual screening efficiencies demonstrated in the blind test set indicate that those multiple biomarkers from MALDI-TOF MS of serum N-glycans were useful for the screening of the ovarian cancer patients, particularly at the early stages.

## Supplementary Material

Supplementary Table 1. A separate set of recurrent ovarian cancer patients was analyzed by MALDI-TOF MS of serum N-glycans as described in the report. Sensitivities for the determination of the screening score thresholds in the training set were indicated (90%, 85%, and 80%). With each threshold, serum samples from recurrent patients were analyzed, and the decisions by the analysis were all patients (P).
Supplementary Table 2. Serum samples in the report were sorted out according to the histological types, and sensitivities of each cell-type were provided. Sensitivities used in the determination of the screening score thresholds were indicated on the top. The sensitivities derived from CA-125 were also demonstrated for comparison.
Supplementary Figure 1. Representative MALDI-TOF MS spectra of 10% ACN/H2O fractions from an ovarian cancer patient (top) and from a disease-free control sample (bottom).
Supplementary Figure 2. Representative MALDI-TOF MS spectra of 20% ACN/H2O fractions from an ovarian cancer patient (top) and from a disease-free control sample (bottom).

## Figures and Tables

**Table 1:** Age distribution of the training set.

Age	Marker discovery set	
	Control	Patient
20~29	6	6
30~39	10	10
40~49	15	15
50~59	9	9

Total	40	40

Average age	41.2 ± 9.7	41.8 ± 9.5

**Table 2:** Stage distribution of the ovarian cancer patients in the training set.

Stage I	19
Stage II	3
Stage III	15
Stage IV	3

Total	40

**Table 3:** Candidate biomarkers with *m*/*z*, detection frequency, mean intensities, *P* values, and AUC in the training set.

Fraction^a^	Marker type	Theoretical *m*/*z*	Measured *m*/*z*		*Δ m*/*z* (10^−3^ Da)	Composition				Frequency		Intensity		*P* value	AUC^c^
			Average	SD^b^ (10^−3^ Da)		Hex	Hex NAc	Fuc	Neu AC	*f*(N)	*f*(P)	Normal	Patient		
10%	N-glycan	1501.5293	1501.5222	3.5	7.1	4	4	0	0	100%	100%	14.28 ± 4.48	10.86 ± 3.76	1.*E* − 12	**0.72 **
		1647.5873	1647.5916	2.2	−4.3	4	4	1	0	100%	100%	70.34 ± 8.91	63.17 ± 9.82	4.*E* − 11	0.71
		1663.5823	1663.5794	2.4	2.9	5	4	0	0	100%	100%	8.93 ± 2.35	6.15 ± 2.13	3.*E* − 24	**0.81 **
		1809.6403	1809.6413	1.8	−1.0	5	4	1	0	100%	100%	9.34 ± 2.25	6.68 ± 3.06	6.*E* − 17	**0.77 **
		1866.6613	1866.6568	2.5	4.5	5	5	0	0	100%	100%	2.03 ± 0.47	1.65 ± 0.42	7.*E* − 14	**0.73 **
		2012.7193	2012.7207	1.6	−1.3	5	5	1	0	100%	100%	9.61 ± 1.85	7.86 ± 1.87	1.*E* − 15	**0.74 **
	Glycan	1467.4853	1467.5312	3.7	−45.9	7	0	2	0	100%	100%	1.15 ± 0.31	1.43 ± 0.43	1.*E* − 10	0.69
	Nonglycan		1384.4855	2.6						100%	100%	1.85 ± 0.64	2.46 ± 0.87	7.*E* − 12	0.71

20%	N-glycan	1444.5073	1444.5009	1.9	6.4	4	3	1	0	100%	100%	3.57 ± 0.40	3.06 ± 0.50	8.*E* − 21	**0.78 **
		1485.5343	1485.5305	1.3	3.8	3	4	1	0	100%	100%	63.17 ± 13.99	78.05 ± 21.98	4.*E* − 12	0.72
		1663.5823	1663.5646	3.6	17.8	5	4	0	0	100%	100%	3.94 ± 0.93	3.08 ± 0.62	5.*E* − 20	**0.82 **
		1809.6403	1809.6399	0.5	0.4	5	4	1	0	100%	100%	98.55 ± 12.93	84.32 ± 20.20	6.*E* − 13	0.72
	Glycan	1483.4803	1483.5146	3.7	−34.3	8	0	1	0	100%	100%	0.86 ± 0.21	1.17 ± 0.49	1.*E* − 12	0.68
		1524.5073	1524.4894	3.8	17.9	7	1	1	0	99%	99%	0.95 ± 0.21	0.72 ± 0.25	4.*E* − 17	**0.76 **
		1540.5023	1540.4550	3.7	47.3	8	1	0	0	100%	100%	2.04 ± 0.58	1.40 ± 0.56	2.*E* − 20	**0.79 **
		1629.5383	1629.5791	3.3	−40.7	8	0	2	0	100%	100%	2.70 ± 0.37	2.39 ± 0.42	4.*E* − 12	0.72
		1791.5913	1791.6336	3.7	−42.3	9	0	1	0	100%	100%	1.48 ± 0.24	1.06 ± 0.32	3.*E* − 32	**0.85 **
	Nonglycan		1280.4357	2.7						100%	100%	2.34 ± 0.36	2.08 ± 0.38	6.*E* − 10	0.69
			1442.4848	3.4						100%	100%	1.21 ± 0.19	0.90 ± 0.23	2.*E* − 30	**0.84 **
			1502.5077	4.8						100%	100%	3.66 ± 0.53	3.15 ± 0.50	1.*E* − 16	**0.75 **
			1518.4722	5.6						100%	98%	0.87 ± 0.37	0.59 ± 0.26	7.*E* − 14	0.75
			1562.4391	7.2						100%	98%	0.67 ± 0.15	0.52 ± 0.14	5.*E* − 19	**0.77 **
			1708.5716	5.5						100%	100%	2.67 ± 0.51	1.93 ± 0.62	1.*E* − 26	**0.82 **
			1945.6596	5.1						100%	100%	1.92 ± 0.30	1.36 ± 0.46	3.*E* − 30	**0.84 **

^a^Acetonitrile/H_2_O fraction in the elution of glycans from graphitized carbon cartridges.

^b^The standard deviation of each *m*/*z* in the training set was calculated and expressed in 10^−3^ Da.

^c^AUC of the biomarkers selected for the use in the later blind test set is in  bold.

**Table 4:** Representative sensitivities and specificities obtained from the training set.

Sensitivity	90%	85%	80%
Specificity	70%	80%	83%
CA-125 sensitivity	74% (cut-off: 35 U/mL)		

Note. Biomarkers used in this classification were denoted in Table 3. Cut-off values of each biomarker were determined according to the standard sensitivity 70%. To combine multiple biomarkers for the screening, a classification score was calculated on the basis of the cut-off values and weighting factors. By varying the thresholds for the classification score, multiple pairs of sensitivity and specificity were acquired from the training set. Sensitivity of CA-125 level was calculated with 35 U/mL as a cut-off value.

**Table 5:** Age distribution of the blind test set.

Age	Blind test	
	Healthy controls	Patients
20~29	4	0
30~39	7	1
40~49	11	13
50~59	1	10
60~69	0	12
70~79	0	0
80~89	0	1

Total	23	37

**Table 6:** Sensitivity and specificity in the blind test set.

Sensitivity (training set)	90%	85%	80%
Sensitivity	84%	84%	81%
Specificity	83%	83%	83%

CA-125 sensitivity	78% (Cut-off: 35 U/mL)		

Note. The multibiomarker panel, cut-off values set by standard specificity 70% (training set), a scoring system with AUC as a weighting factor, and sensitivities (top) for the thresholds of scores in the training set were identical with Table 4 except that the blind test set was evaluated in Table 6.
