# Supplementary material for: Mass Spectrometric Screening of Ovarian Cancer with Serum Glycans
Source: Dis Markers. 2014 Feb 4;2014:634289. doi: 10.1155/2014/634289 (PMC3932261; doi:10.1155/2014/634289)
Supplement: Supplementary file 1 — Supplementary Table 1. A separate set of recurrent ovarian cancer patients was analyzed by MALDI-TOF MS of serum N-glycans as described in the report. Sensitivities for the determination of the screening score thresholds in the training set were indicated (90%, 85%, and 80%). With each threshold, serum samples from recurrent patients were analyzed, and the decisions by the analysis were all patients (P). Supplementary Table 2. Serum samples in the report were sorted out according to the histological types, and sensitivities of each cell-type were provided. Sensitivities used in the determination of the screening score thresholds were indicated on the top. The sensitivities derived from CA-125 were also demonstrated for comparison. Supplementary Figure 1. Representative MALDI-TOF MS spectra of 10% ACN/H2O fractions from an ovarian cancer patient (top) and from a disease-free control sample (bottom). Supplementary Figure 2. Representative MALDI-TOF MS spectra of 20% ACN/H2O fractions from an ovarian cancer patient (top) and from a disease-free control sample (bottom). [file 634289.f1.pdf]

Supplementary Table 1. Successful classification of recurrent ovarian cancer patients

Age	Stage	Cell type	Recurrence	CA125 (U/ml)	Screening (P: patient)		
					90%[a]	85%[a]	80%[a]
50	Ia	SCC	Y		P	P	P
42	Ic	serous cystadenocarcinoma	Y		P	P	P
54	IIa, G2	adenocarcinoma, mixed endometrioid	Y	68.55	P	P	P
56	IIIa, G3	serous adenocarcinma	Y	81.3	P	P	P
67	IIIc	strumal carcinoid	Y		P	P	P
58	IIIc, G3	serous adenocarcinma	Y	33.51	P	P	P
41	IIIc, G3	mixed serous and transitional cell carcinoma	Y	27.69	P	P	P
58	IV	MMMT	Y	82.49	P	P	P

[a] Sensitivities for the determination of the screening score thresholds in the training set. In the following column, the same score threshold was used for the screening of the test samples.

Supplementary Table 2. Classification of ovarian cancer sera depending on the cell types

Sensitivity standard for threholds (Training set)		90%	85%	80%	CA-125
Sensitivity in the training set	Clear cell type	100%	100%	100%	80%
	Serous type	88%	81%	69%	73%
Sensitivity in the blind test set	Clear cell type	89%	89%	89%	67%
	Serous type	93%	93%	87%	92%

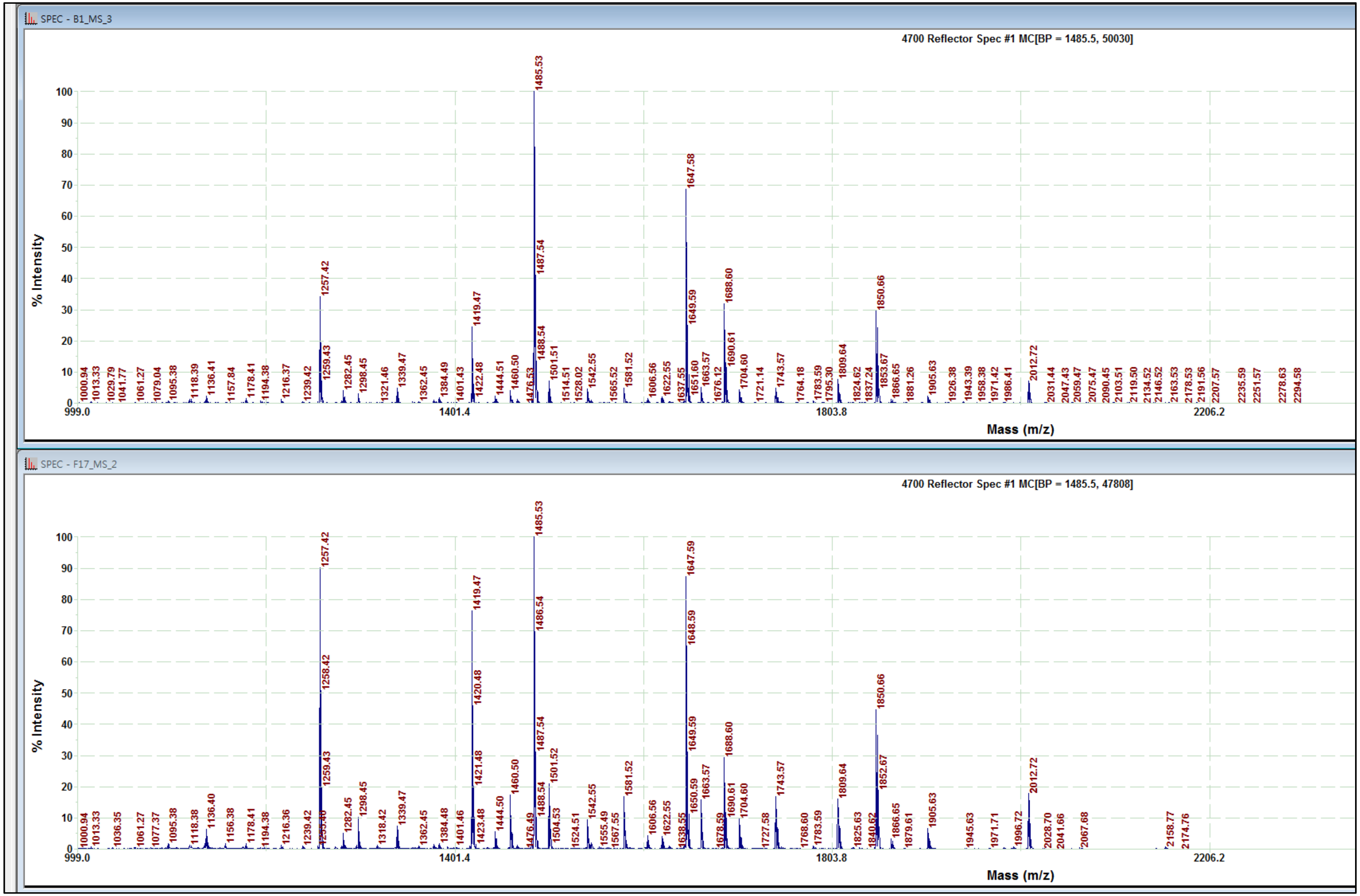

Supplementary Figure 1

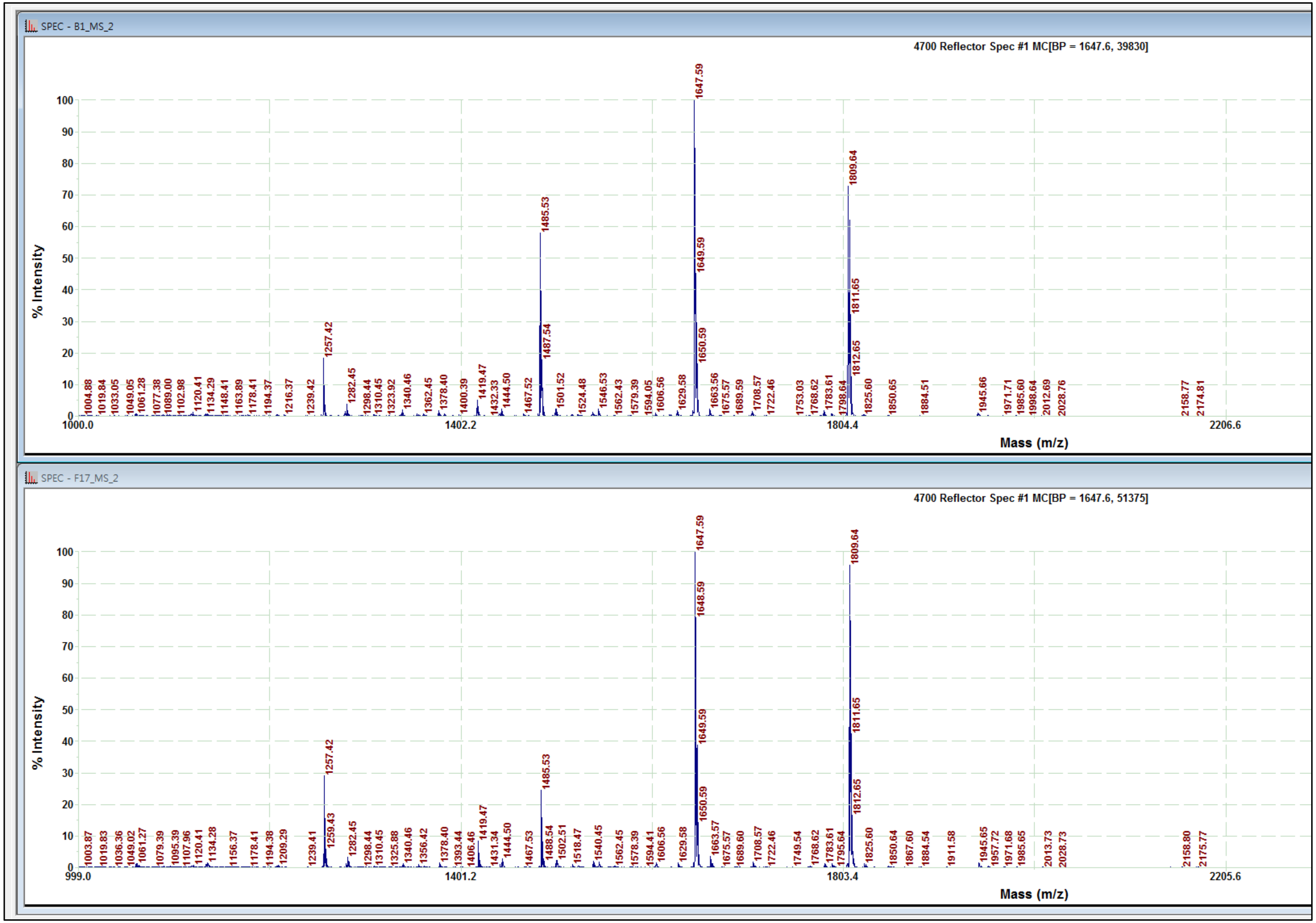

Supplementary Figure 2
